# MiR‐20a‐5p Inhibits Bladder Cancer Proliferation and Migration by Targeting KPNA2

**DOI:** 10.1111/jcmm.70785

**Published:** 2025-08-19

**Authors:** Shuai Ye, Cen Liufu, Cong Yin, Tao Zhu, Jinqing He, Yuanyuan Tian, Yan Wang, Bentao Shi

**Affiliations:** ^1^ Department of Urology The First Affiliated Hospital of Shenzhen University, Shenzhen Second People's Hospital Shenzhen China; ^2^ Shenzhen University Health Science Center Shenzhen China; ^3^ Shantou University Medical College Shantou China; ^4^ Department of Urology Peking University Shenzhen Hospital, Institute of Urology, Shenzhen PKU‐HKUST Medical Center Shenzhen China; ^5^ Central Laboratory Shenzhen Qianhai Taikang Hospital Shenzhen China

**Keywords:** bladder cancer, KPNA2, migration, MiR‐20a‐5p, proliferation

## Abstract

Bladder cancer (BC) is one of the 10 most common cancers in the world, and its recurrence and metastasis are the main causes of death in BC patients. Exploring the molecular mechanisms of BC pathogenesis and searching for new prognostic markers and therapeutic targets are important for improving patient prognosis. KPNA2 was found to be a potential oncogene in different malignant tumours, as demonstrated in our previous study. To better understand the mechanisms associated with BC development, we investigated the inhibitory effect of miR‐20a‐5p on the oncogene KPNA2. RNA‐seq data from BC patients were downloaded through the TCGA database for bioinformatics analysis, including gene expression, co‐expression analysis, GSEA, nomogram modelling, functional enrichment analysis, WGCNA, GO and KEGG to assess the potential biological functions of miR‐20a‐5p in BC. Subsequently, we further verified the expression of miR‐20a‐5p in BC cells by RT‐qPCR, and in vitro experiments were performed to investigate the effects of this gene on BC cell proliferation and migration. MiR‐20a‐5p was downregulated in BC tissues and cells. Kaplan–Meier analysis revealed that the higher the expression of miR‐20a‐5p in patients, the higher the survival rate of BC patients. MiR‐20a‐5p overexpression inhibited the proliferation and migration of BC cells. In addition, miR‐20a‐5p can directly bind to nuclear transporter protein α2 (KPNA2) in cells, targeting and regulating the expression of KPNA2. These findings indicate that miR‐20a‐5p targeting KPNA2 adversely affects the proliferation and migration of BC cells, suggesting that miR‐20a‐5p may be an attractive target in BC therapy.

## 1 Introduction

Bladder cancer (BC), as a highly prevalent malignant tumour of the urinary system worldwide, has recently called for extensive attention and in‐depth exploration in both clinical and basic research. According to epidemiological data, the number of new BC cases globally exceeded 610,000 in 2022, with related deaths reaching as high as 220,000, profoundly threatening global public health [1]. Although comprehensive treatment strategies centred on surgical intervention have significantly improved patient survival rates, and adjuvant chemotherapy and other treatments continue to evolve, the high recurrence rate and propensity for metastasis of BC still pose major challenges in clinical management, seriously affecting patient prognosis and quality of life [2]. Therefore, an in‐depth investigation of the molecular mechanisms of BC, especially its biological processes involved in initiation, progression and malignant development, has become the scientific foundation for achieving precise diagnosis and targeted therapy.

In this context, microRNAs (miRNAs), as key members of non‐coding RNAs, and their roles in tumours have gradually become a research hotspot in recent years. miRNAs are a class of endogenous non‐coding small RNAs approximately 19–25 nucleotides in length, primarily regulating gene post‐transcriptional expression by binding to the 3′ untranslated region (3′UTR) of target gene mRNAs, thereby influencing cell growth and function [3]. A substantial body of research evidence indicates that miRNAs play an irreplaceable central role in biological processes such as cell proliferation, apoptosis, differentiation, migration and immune response [4]. Moreover, the abnormal expression of miRNAs has been found to be closely associated with various human diseases, particularly the occurrence, progression and therapeutic resistance of multiple types of cancer [5, 6, 7]. The latest systematic reviews and database updates have conducted in‐depth identification of disease‐related miRNAs and their potential targets, which not only provide a new perspective for understanding the important role of miRNAs in disease pathogenesis but also offer a practical basis for utilising miRNAs in early diagnosis and therapeutic design [8, 9].

With the rapid development of high‐throughput omics technologies and the widespread application of bioinformatics analysis tools, the expression profiles of miRNAs and their associated target gene networks have been revealed in unprecedented depth, providing a solid molecular foundation for early cancer diagnosis, disease subtype monitoring and the formulation of personalised treatment strategies. Advances in these technologies have enabled us to fully explore the complex relationships between miRNAs and human diseases, promoting the in‐depth development of miRNA–disease association (MDA) research. Researchers have recognised the necessity of shifting the study of miRNA–disease relationships from traditional laboratory exploration to a bioinformatics approach that integrates multi‐source data and complex algorithmic models. This new data integration paradigm allows researchers to more efficiently identify functional miRNAs related to tumorigenesis and progression, thereby accelerating the progress of MDA research [10].

miR‐20a‐5p is considered an important member of the miR‐17~92 gene cluster, and its biological functions and clinical application value have increasingly attracted attention [11]. Existing literature shows that miR‐20a‐5p not only participates in regulating fundamental biological behaviours such as proliferation, apoptosis and invasion of various tumour cells, but its specific expression pattern is also closely associated with the development of multiple malignant tumours [12, 13, 14]. Therefore, it has been proposed as a potential tumour diagnostic biomarker or therapeutic target. In studies of prolactinomas and certain haematological tumours, miR‐20a‐5p has become a key focus in the field of liquid biopsy due to its unique expression characteristics and the advantage of non‐invasive detection, marking its growing recognition for potential in tumour diagnosis and treatment [15]. Notably, miR‐20a‐5p can exhibit a dual role as either an oncogene or tumour suppressor depending on the tumour context. For example, in endometrial cancer, the expression of miR‐20a‐5p is significantly downregulated, and it inhibits epithelial–mesenchymal transition (EMT) and invasion by targeting STAT3 [16]. In neuroblastoma, miR‐20a‐5p promotes apoptosis by targeting autophagy‐related genes such as ATG7 [17]. Meanwhile, in gastric cancer cells, this miRNA promotes tumour cell proliferation, migration and invasion by downregulating WTX, thereby further activating the PI3K/AKT pathway [18]. These findings not only deeply reveal the complexity of the gene regulatory network mediated by miR‐20a‐5p but also expand its potential diverse biological functions and clinical application prospects.

Karyopherin subunit alpha 2 (KPNA2), as a member of the karyopherin family, plays a crucial role in intracellular transport and transcriptional regulation. Studies have shown that KPNA2 interacts with the nuclear localisation sequences (NLS) of proteins such as SV40 large T antigen and Myc [19, 20]. Aberrant expression of KPNA2 is closely associated with the occurrence and progression of various cancers, especially gallbladder cancer, colorectal cancer and breast cancer, where its pro‐tumorigenic properties have been widely confirmed [21, 22, 23]. As a potential cancer biomarker, KPNA2 promotes tumorigenesis by affecting cell differentiation, proliferation and apoptosis. However, the relationship between miR‐20a‐5p and KPNA2 in BC remains unclear. Given the contradictory reports regarding the role of miR‐20a‐5p in different malignancies, this study aims to systematically investigate the expression of KPNA2 and miR‐20a‐5p in BC and their regulatory mechanisms. Through this research, we hope to gain a deeper understanding of the molecular characteristics of BC and provide new potential targets and theoretical support for miRNA‐based targeted therapies. The study will focus on the interaction between miR‐20a‐5p and KPNA2, employing a comprehensive approach that includes bioinformatics analysis, comparative tissue sample studies and functional experiments to thoroughly evaluate the role of this molecular axis in cell cycle regulation and tumour invasion and metastasis. Through this work, we aim to clarify the molecular mechanisms of BC, promote the development of related targeted therapeutic strategies and provide a solid theoretical foundation for precise clinical interventions to more effectively address this complex disease.

## 2 Materials and Methods

### 2.1 TCGA Data Acquisition and Analysis

The Cancer Genome Atlas (TCGA) database (https://portal.gdc.cancer.gov/) is one of the largest repositories of cancer genomic information, containing data on gene expression, copy number variations and single nucleotide polymorphisms (SNPs), among others. We downloaded raw mRNA expression data and SNP data of BC, which include 19 normal samples and 412 tumour samples, to analyse miRNA expression differences, prognosis and specific regulatory mechanisms.

### 2.2 Co‐Expression Analysis

Pearson correlation analysis was performed to analyse the co‐expression of the miR‐20a‐5p gene in BC data. The correlation coefficient filtering condition was set at 0.4 with a *p* value of 0.05. After selecting the genes that showed the most significant correlation with miR‐20a‐5p expression, ‘(corrplot 0.92)’ and ‘(circlize 0.4.15)’ packages were utilised to generate correlation analysis circular plots and heatmaps for miR‐20a‐5p.

### 2.3 Diagnostic and Prognostic Value Analysis

The receiver operating characteristic (ROC) curve is a method used to evaluate the performance of diagnostic tests, while the time‐dependent ROC curve provides information on diagnostic efficacy over time. Both are among the most commonly used tools for estimating and analysing the diagnostic and prognostic value of patients. In the TCGA‐BLCA dataset, the diagnostic capability of the miR‐20a‐5p gene was analysed, and subsequently, the ‘(timeROC 0.4)’ package was employed to generate ROC and time‐dependent ROC curves. The Kaplan–Meier (KM) survival curve is used to analyse patient survival probability. Following data collection, survival analysis was conducted using the ‘(survminer 0.4.9)’ package, and the KM curve was plotted. *p* < 0.05 was considered statistically significant.

### 2.4 Gene Set Enrichment Analysis (GSEA)

Based on the expression levels of miR‐20a‐5p, patients were divided into high and low‐expression groups. The ‘(GSEA 4.3.2)’ package was subsequently employed to analyse these two groups' differential signalling pathway enrichment. The background gene set was downloaded from the MsigDB database (version 7.0). Significantly enriched gene sets (adjusted *p* value < 0.05) were ranked based on enrichment scores.

### 2.5 Nomogram Model Construction

Nomogram is a tool based on regression analysis that utilises gene expression levels and clinical symptoms. A predictive model employs scaled line segments drawn on the same plane according to a certain proportion to represent the relationships between variables. By constructing a multiple regression model, the contribution of each influencing factor to the outcome variable (reflected by the magnitude of regression coefficients) is assessed. Scores are assigned to each level of the influencing factors, and these scores are then summed to obtain a total score, which is used to calculate the predicted value. Nomogram enables us to understand better and apply predictive models, providing accurate predictions and facilitating personalised medical decision‐making based on gene expression and clinical information.

### 2.6 Construction of the Weighted Gene Co‐Expression Network Analysis (WGCNA) and Functional Enrichment Analysis

The ‘(WGCNA 1.72‐1)’ package constructs a co‐expression network of all genes in the BC dataset, screening the top 5000 genes in variance with this algorithm for further analysis, with the soft threshold set to 9. The weighted neighbour‐joining matrix is transformed into a topological overlap matrix (TOM) to estimate the degree of network connectivity. The hierarchical clustering method is applied to construct a clustering tree structure for the TOM matrix. The genes are grouped into modules according to their expression patterns based on the weighted correlation coefficients of genes. In order to classify the genes according to their expression patterns, the genes with similar patterns were grouped into one module, and all the genes were classified into multiple modules by their gene expression patterns. Functional annotation of the modular genes was performed using the R package ‘(ClusterProfiler 4.6.2)’ to explore the functional relevance of these modular genes comprehensively. Gene Ontology (GO) and Kyoto Encyclopedia of Genes and Genomes (KEGG) were used to assess the relevant functional categories. *p* values and *q* values of less than 0.05 for GO and KEGG‐enriched pathways were considered significant.

### 2.7 Screening of miR‐20a‐5p Targeted Regulatory Genes

To explore the impact of miR‐20a‐5p on tumorigenesis and progression at the molecular level, we utilised the online prediction tool miRDB (https://mirdb.org/) to identify genes targeted by miR‐20a‐5p.

### 2.8 Cell Lines and Cell Cultures

The human embryonic kidney epithelial cell line HEK293T, human BC cell lines (T24, J82, UMUC‐3 and SW780) and the normal bladder epithelial cell line SV‐HUC‐1 were obtained from the American Type Culture Collection (ATCC). The cells were cultured in the recommended medium supplemented with 10% fetal bovine serum (FBS) and 100 U/mL penicillin/streptomycin at 37°C with 5% CO_2_.

### 2.9 RNA Extraction and RT‐qPCR

Total RNA was extracted from samples using the TRIzol reagent (Takara, Japan). For each sample, 1 μg of total RNA was reverse‐transcribed using the PrimeScript RT Reagent Kit with gDNA Eraser (Takara, Japan), specifically designed for RT‐qPCR. Quantitative PCR (qPCR) amplification was then performed on a LightCycler 480 system (Roche, USA) using the SYBR Premix Ex Taq II Kit (Takara, Japan). GAPDH was used as the reference gene, and the relative expression levels were calculated using the 2^−ΔΔCt^ method. The primer sequences were synthesised by a biotech company (Shanghai, China) and are as follows:

GAPDH‐F: CCACTCCTCCACCTTTGACG;

GAPDH‐R: CTGGTGGTCCAGGGGTCTTA;

miR‐20a‐5p: GGCTAAAGTGCTTATAGTGCAGGTAG.

### 2.10 Dual‐Luciferase Reporter Assay

The dual‐luciferase reporter assay is a widely used experimental technique for validating the direct interaction between miRNAs and their target genes. By constructing a dual‐luciferase reporter vector containing the KPNA2‐3'UTR and co‐transfecting it with miR‐20a‐5p mimics into cells, the binding ability of miR‐20a‐5p to the KPNA2‐3'UTR can be assessed. If miR‐20a‐5p directly binds to the KPNA2‐3'UTR, it is expected to inhibit luciferase activity, paracancerous thereby reducing the reporter signal.

### 2.11 MiRNA Mimics Transfected BC Cell Lines

BC cell lines were seeded at a density of 10^5^ cells per well in six‐well plates and incubated at 37°C with 5% CO_2_ until reaching 50%–60% confluence, at which point the culture medium was removed for transfection. The cells were then washed with phosphate‐buffered saline (PBS) and supplemented with an appropriate amount of serum‐free medium. For each well, the following transfection mixture was prepared: 10 μL miRNA mimics, 100 μL Opti‐MEM and 8 μL Lipofectamine RNAiMAX transfection reagent. The mixture was gently flicked to ensure even distribution and left to incubate at room temperature for 5 min to allow complex formation. After adding the transfection mixture to the culture wells, the plate was gently shaken for uniform distribution and left undisturbed for 15–30 min. Following transfection, the BC cells were incubated at 37°C with 5% CO_2_ for 4–6 h, after which the medium was replaced with complete culture medium. The cells were then further incubated for 24–48 h to assess the effects of miRNA mimics. The miRNA mimics were synthesised by GenePharma (China), with the following sequences:

hsa‐miR‐20a‐5p mimics:

UAAAGUGCUUAUAGUGCAGGUAG;

ACCUGCACUAUAAGCACUUUAUU.

### 2.12 CCK‐8 Assay

BC cells were plated in 96‐well plates at a density of 1.5× 10^3^ cells per well, with 100 μL of medium. The plates were covered with aluminium foil and incubated for 5–6 h until the cells adhered. At 0, 24, 48 and 72 h post‐transfection, 10 μ L of Cell Counting Kit‐8 (CCK‐8) reagent was added to each well under dark conditions. Following a 3‐h incubation, the plates were placed in a microplate reader, and the optical density (OD value) at 450 nm was measured and recorded.

### 2.13 Wound Healing Assay

Cells were seeded in six‐well plates and allowed to adhere. A straight scratch wound was carefully made using a 20‐μL pipette tip, and the plate was washed with PBS to eliminate any cellular debris. Afterwards, 2 mL of serum‐free medium was added to the wells. The width of the wound was measured after 24 h using an inverted microscope, and the images were analysed using Photoshop software.

### 2.14 Cell Cycle Assays

BC cells digested with trypsin were collected, centrifuged and washed twice with PBS. The cells were then fixed with pre‐chilled 70% ethanol at 4°C for 24 h. Subsequently, the cells were stained with propidium iodide/RNase staining buffer (BD Biosciences) at room temperature for 30 min in the dark. After staining, the cells were analysed using flow cytometry; data analysis was performed with FlowJo V10 software. All experiments were conducted in triplicate.

### 2.15 Statistical Analysis

All statistical analyses were performed using R language (version 4.3.0), with *p* < 0.05 considered statistically significant. Experimental results were analysed using GraphPad Prism 9, and data were presented as mean ± standard deviation (SD), with experiments repeated at least three times. Comparisons between groups were conducted using the independent sample *t*‐test and one‐way analysis of variance (ANOVA).

## 3 Results

### 3.1 MiR‐20a‐5p Is Lowly Expressed in BC Tissues

**FIGURE 1:**
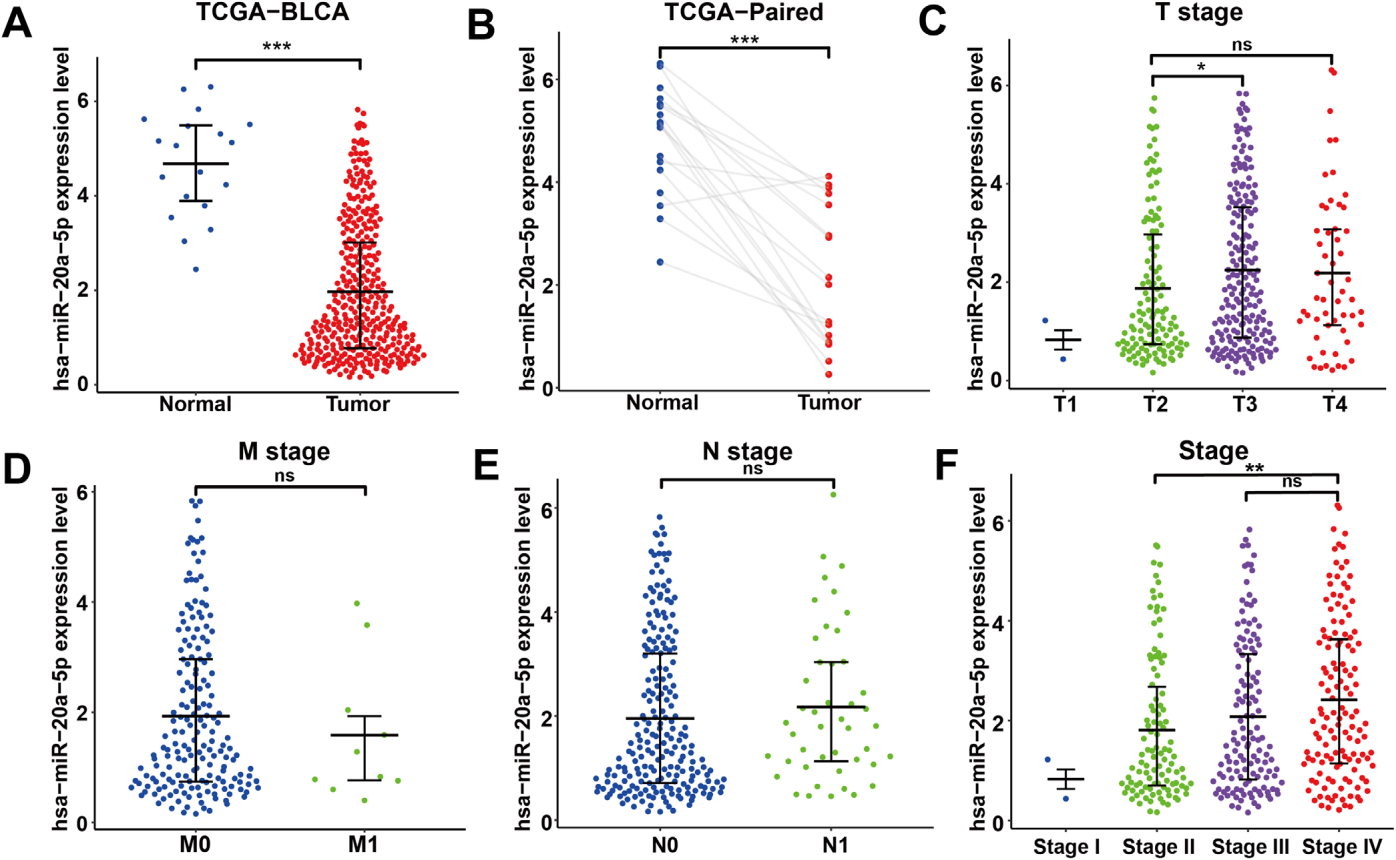
Association between miR‐20a‐5p expression and clinicopathological features of BC. (A) Expression levels of miR‐20a‐5p in BC and normal tissues based on TCGA data. (B) Comparative expression of miR‐20a‐5p between matched normal and tumour tissues from the same samples. (C) Expression of miR‐20a‐5p in the T stage of BC. (D) Expression of miR‐20a‐5p in the M stage of BC. (E) Expression of miR‐20a‐5p in the N stage of BC. (F) Expression of miR‐20a‐5p in the tumour Stage of BC. **p < 0.01; ***p < 0.001; ns, not significant.

The specific mechanism of miR‐20a‐5p in BC remains unclear. We downloaded and integrated miRNA data of BC from the TCGA database, which revealed differential expression of miR‐20a‐5p between normal and tumour tissues (Figure 1A), with a significant downregulation of miR‐20a‐5p in tumour tissues. Additionally, in tumour tissues of the same samples, miR‐20a‐5p expression was lower than in normal tissues (Figure 1B), further suggesting that the low expression of miR‐20a‐5p may induce the onset of tumours. Tumour staging refers to the extent of malignant tumour growth and dissemination. The larger the tumour volume, the wider the growth and dissemination range, leading to poorer prognosis. Staging involves considering factors such as the size of the primary tumour, depth of invasion, extent of invasion, involvement of adjacent organs, local and distant lymph node metastasis and distant metastasis. By analysing the relationship between miR‐20a‐5p and BC staging in the TCGA dataset, we further investigated the correlation between this gene and BC progression. The results indicated that as the primary tumour size increased and the extent of adjacent tissue involvement grew (T stage), the expression of miR‐20a‐5p initially increased before decreasing (Figure 1C). However, miR‐20a‐5p expression showed a marked decrease with distant metastasis (M stage) and local lymph node infiltration (N stage) (Figure 1D,E), suggesting that this gene might serve as a potential target for monitoring tumour metastasis. Finally, based on the expression changes of miR‐20a‐5p at different stages of BC, we observed that even in high‐grade tumours, there was no significant change in its expression. Nevertheless, TCGA data revealed that miR‐20a‐5p expression was elevated compared to low‐grade tumours (Figure 1F). This phenomenon may indicate that the regulation of miR‐20a‐5p in tumour tissues is more likely a compensatory and decompensatory mechanism under a negative feedback control system. Specifically, the low expression in the early stages may provide certain advantages for the growth and spread of tumour cells. In the later stages, when tumour cells increase in size and energy consumption rises, the upregulation of miR‐20a‐5p expression may serve to maintain the stability of the intracellular environment and prevent further aggravation of malignant transformation. This indicates that miRNAs play regulatory roles at different stages of tumours. These changes are not only a response to tumour development but also the result of the complex regulatory networks within the cells.

### 3.2 Diagnostic and Prognostic Value Analysis of miR‐20a‐5p

**FIGURE 2:**
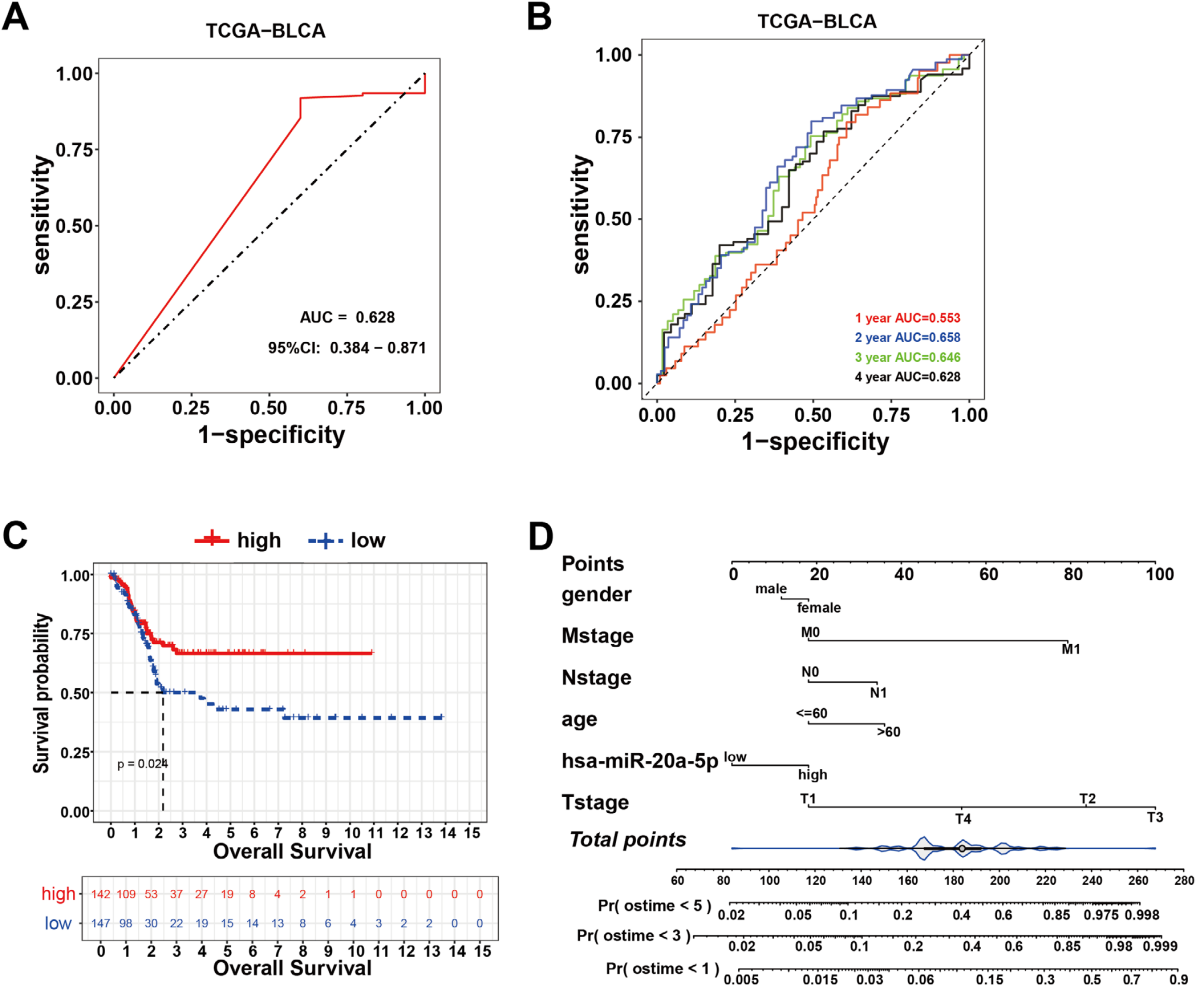
Patients with high miR‐20a‐5p expression have a better prognosis. (A) ROC curve of miR‐20a‐5p in TCGA. (B) Time‐dependent ROC curve of miR‐20a‐5p in TCGA. (C) Kaplan–Meier survival curve of miR‐20a‐5p. (D) Prognosis‐related Nomogram model of miR‐20a‐5p.

The receiver operating characteristic (ROC) curve is one of the most commonly used evaluation metrics for disease diagnosis. Therefore, we analysed the diagnostic performance of the miR‐20a‐5p in the TCGA‐BLCA dataset. The results showed that the area under the ROC curve (AUC) was 0.628, with a 95% confidence interval of 0.384–0.871 (Figure 2A), indicating that miR‐20a‐5p has a certain capability in distinguishing BC patients from non‐patients. Time‐dependent ROC curves provide a more detailed assessment of the prognostic ability of miR‐20a‐5p at different time points. Notably, the gene exhibited the strongest diagnostic performance at the 2‐year time point (with the highest AUC), while its diagnostic ability was the weakest at the 1‐year time point (Figure 2B).

Kaplan–Meier (KM) survival curves were utilised to assess the prognostic significance of miR‐20a‐5p, with the median expression level serving as the reference value. The results demonstrated that the survival rate of the high miR‐20a‐5p expression group (solid red line) was significantly higher than that of the low expression group (dashed blue line), with a *p* value of 0.024 (Figure 2C), suggesting that high miR‐20a‐5p expression is associated with better overall survival.

Furthermore, to illustrate how integrating miR‐20a‐5p expression with other variables could enhance the accuracy of prognostic predictions, we constructed a prognosis‐related nomogram model based on the TCGA‐BLCA dataset by incorporating miR‐20a‐5p expression levels and other commonly used clinical characteristics. In this model, patients with older age, higher TNM stage and higher miR‐20a‐5p expression had higher scores, which corresponded to shorter survival times (Figure 2D). The nomogram model, in addition to expanding the analysis of Figure 2C, serves as a comprehensive prognostic prediction tool by integrating multiple variables.

### 3.3 MiR‐20a‐5p Is Steadily Low Expression in BC Clinical Samples

**FIGURE 3:**
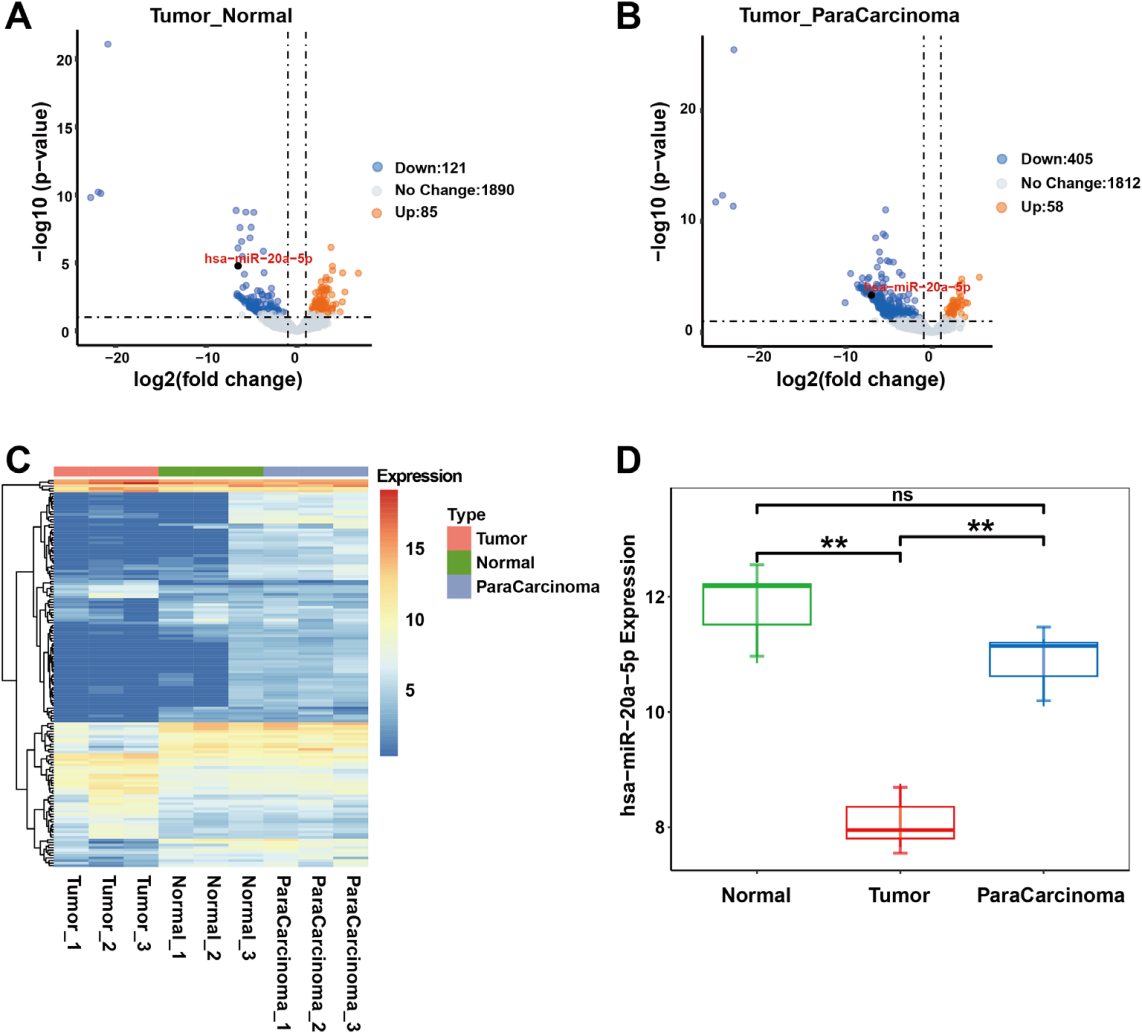
miR‐20a‐5p is lowly expressed in BC clinical samples. (A) MiRNA differential gene volcano plot of BC tumour and normal tissues. (B) MiRNA differential gene volcano plot of BC tumour and paracancerous tissues. (C) MiRNA differential gene heat map of tumour, normal and paracancerous tissues. (D) Box plots of miR‐20a‐5p expression in tumour, normal and paracancerous tissues. **p < 0.01; ns, not significant.

To validate the findings from the TCGA data analysis, this study further examined the miRNA expression data of BC patients from the GEO database (GSE236933, including 25 normal samples and 38 tumour samples). The samples were categorised into three groups: tumour tissue, paracancerous tissue and normal tissue. Both the volcano plot and the heatmap of gene expression revealed that, in differential expression analysis, miRNAs represented by miR‐20a‐5p were significantly downregulated in tumour tissues compared to both paracancerous tissues and normal tissues, with |log2FC| > 1 and *p* value < 0.05 considered significantly upregulated or downregulated (Figure 3A–C). Similarly, a box plot of miR‐20a‐5p expression was used to quantify these differences (Figure 3D). The integration of these data provides comprehensive evidence that miR‐20a‐5p is downregulated in BC, a finding consistent with the results of the TCGA analysis.

### 3.4 Identification of miR‐20a‐5p Target Genes and Analysis of Regulatory Networks

**FIGURE 4:**
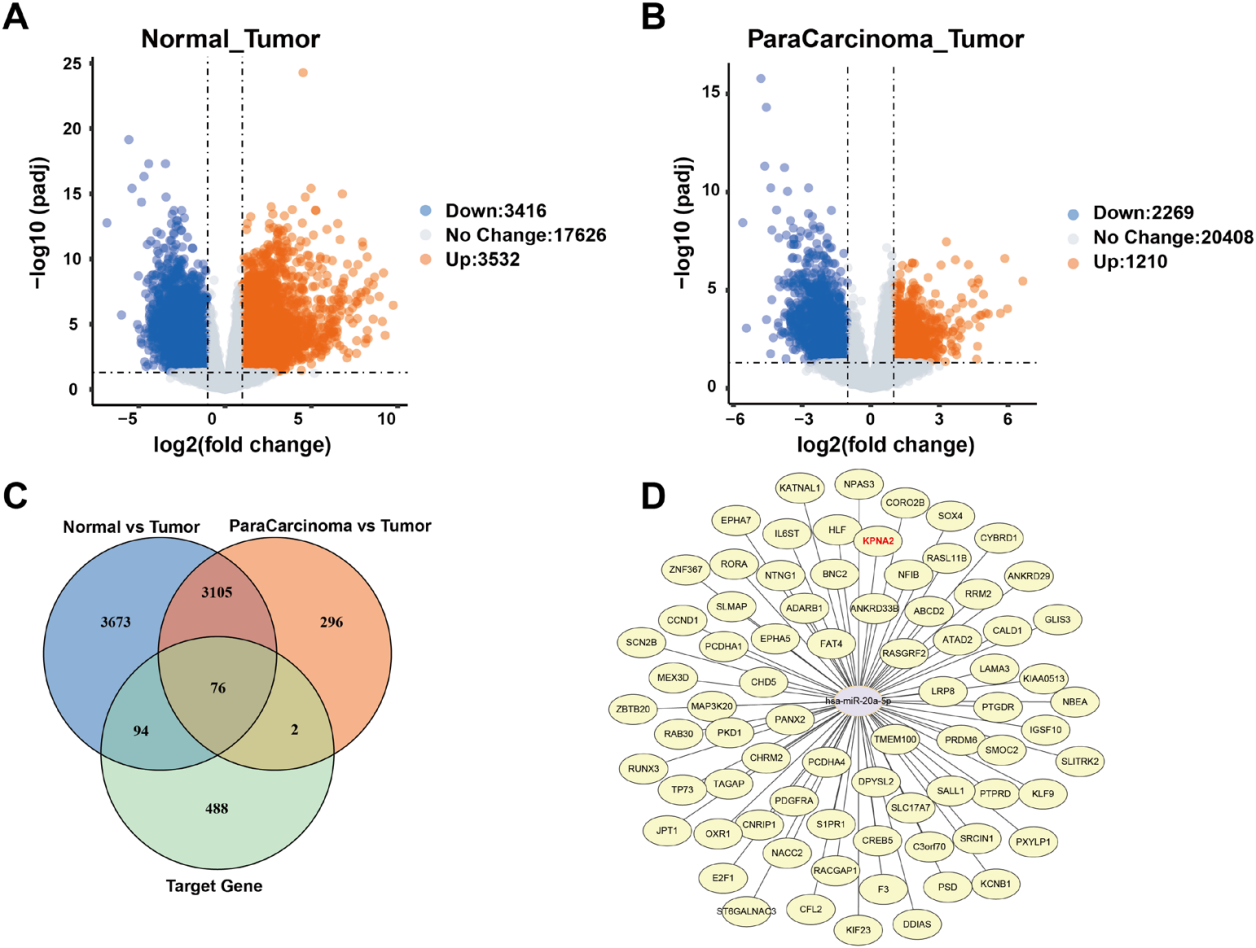
Relevant target genes regulated by miR‐20a‐5p. (A) Volcano plot of mRNA differential genes in BC tumour and normal tissues. (B) Volcano plot of mRNA differential genes in BC tumour and paracancerous tissues. (C) Wenn plots of mRNA differential genes and miR‐20a‐5p target genes. (D) Target gene network diagram of miR‐20a‐5p.

In cancer, miRNAs primarily exert their regulatory functions by modulating the mRNA of their corresponding target genes, thereby influencing cancer progression. Therefore, we further analysed the differential expression of mRNAs in cancer tissues compared to normal or paracancerous tissues. Compared with normal tissues, a total of 3416 downregulated genes and 3532 upregulated genes were identified, with |log2FC| > 1 and *p* value < 0.05 considered significant upregulation/downregulation (Figure 4A). Compared with paracancerous tissues, 2269 downregulated genes and 1210 upregulated genes were identified (Figure 4B). To further illustrate the overlap of differentially expressed genes among different comparison groups, we identified the target genes of miR‐20a‐5p using the miRDB database (https://mirdb.org/) with a filtering criterion of Target Score > 80, yielding 660 reliably scored target genes. These target genes were intersected with the differentially expressed genes to generate a Venn diagram, ultimately identifying 76 target genes of miR‐20a‐5p involved in the regulation of BC (Figure 4C). Other differentially expressed genes may reflect distinct biological processes occurring during tumour progression. To better understand the interaction network of these differentially expressed genes and their molecular‐level impact on tumorigenesis and progression, we constructed a miR‐20a‐5p‐target gene interaction network (Figure 4D) to elucidate the potential molecular mechanisms underlying tumour development.

### 3.5 Gene Enrichment Analysis

**FIGURE 5:**
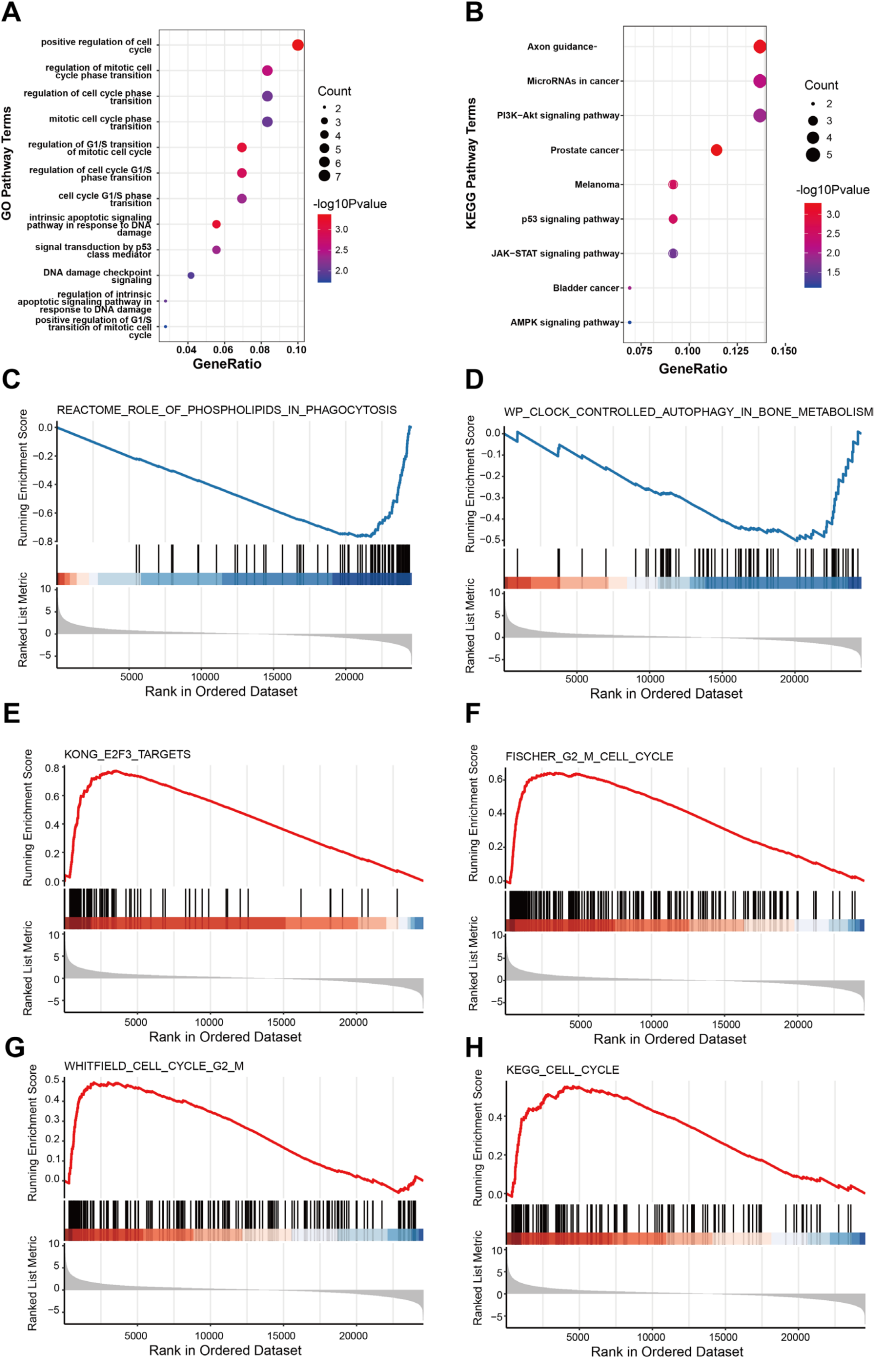
Specific signalling pathways involved in miR‐20a‐5p. (A) GO enrichment analysis. (B) KEGG enrichment analysis. (C–H) GSEA enrichment analysis.

To further explore the potential molecular mechanisms by which miR‐20a‐5p influences BC progression, we performed functional enrichment analysis on the 76 target genes to identify specific signalling pathways associated with miR‐20a‐5p. The results of GO enrichment analysis and KEGG pathway analysis (Figure 5A,B) revealed that these genes were primarily enriched in pathways related to the cell cycle, DNA damage response and p53 signalling. This suggests that miR‐20a‐5p may suppress tumour progression by regulating its target genes to inhibit cell proliferation and block cell cycle progression.

Moreover, GSEA analysis indicated that patients with high miR‐20a‐5p expression were also significantly enriched in cell cycle‐related pathways (Figure 5C–H), which is consistent with the GO and KEGG analyses. Additionally, GSEA results demonstrated an association between miR‐20a‐5p target genes and biological processes such as autophagy, suggesting that miR‐20a‐5p may inhibit BC progression by modulating its target genes to induce autophagy in cancer cells.

These findings complement each other, contributing to a comprehensive understanding of the molecular mechanisms underlying BC development and potentially providing new therapeutic targets for future treatment strategies.

### 3.6 MiR‐20a‐5p Targets and Regulates KPNA2

**FIGURE 6:**
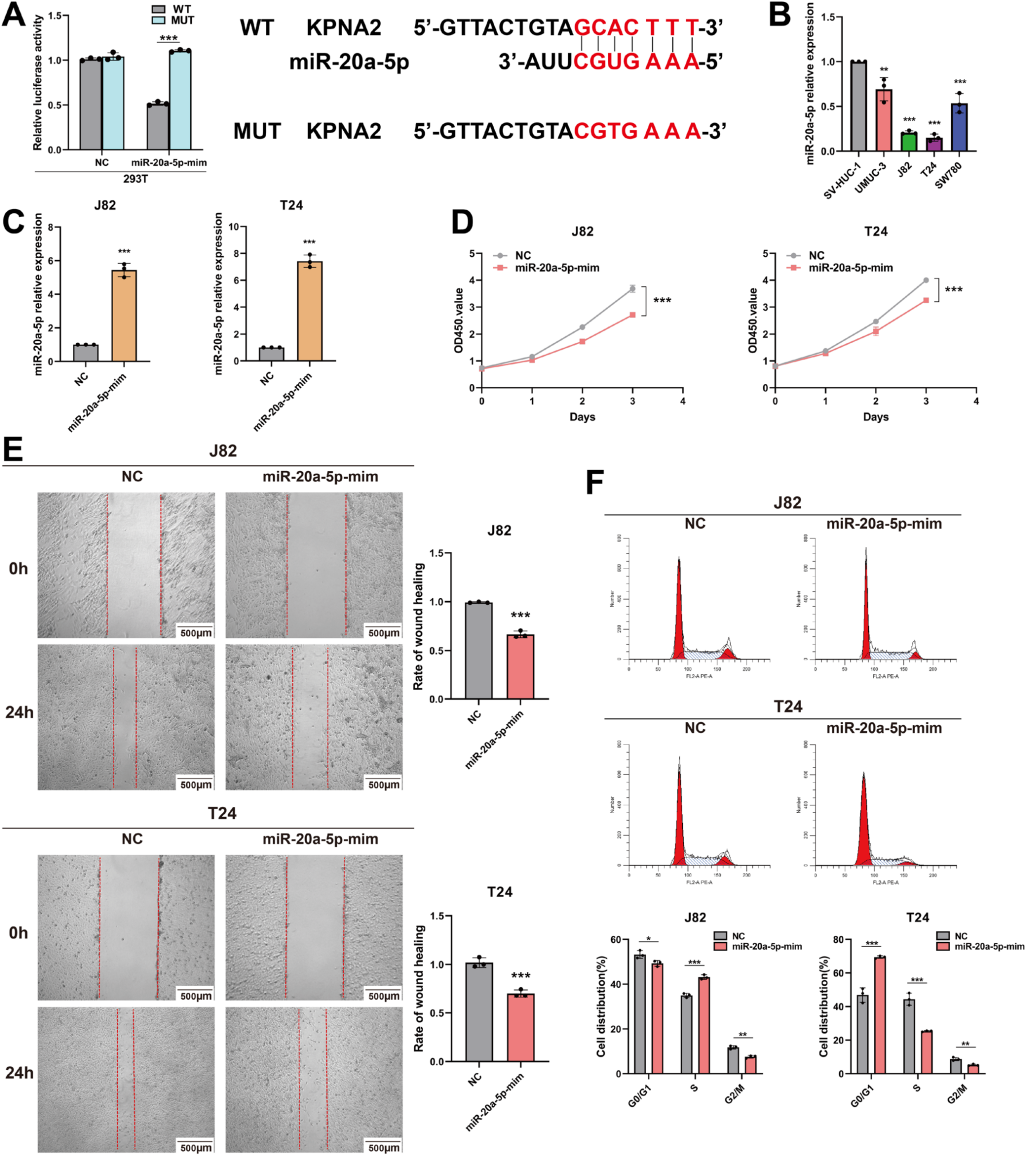
In vitro experiments confirm the tumour suppressor effect of miR‐20a‐5p in BC. (A) Dual luciferase reporter assay confirms the targeted regulatory effect of miR‐20a‐5p on KPNA2. (B) RT‐qPCR analysis of miR‐20a‐5p expression in BC cell lines. (C) Verification of overexpression efficiency of miR‐20a‐5p‐mimics by RT‐qPCR. (D) CCK‐8 assay demonstrated that miR‐20a‐5p was able to reduce the proliferation ability of BC cells. (E) Wound healing assay showed that miR‐20a‐5p was able to inhibit the migration ability of BC cells. (F) Flow cytometry analysis showed that miR‐20a‐5p was able to block the cell cycle progression of BC cells. **p < 0.01; ***p < 0.001; NC, negative control.

To further investigate the molecular mechanisms underlying the biological function of miR‐20a‐5p in BC, we utilised bioinformatics analysis to predict 76 potential target genes regulated by miR‐20a‐5p (Figure 4D). Among these target genes, KPNA2 has been previously identified as an oncogene in BC [19, 24]. To explore the relationship between miR‐20a‐5p and KPNA2 in BC, we performed a dual‐luciferase reporter assay. Firefly luciferase was used as an internal control to ensure the accuracy of the results. The result confirmed that miR‐20a‐5p inhibits KPNA2 expression by directly targeting its 3′ UTR (Figure 6A).

### 3.7 Overexpression of miR‐20a‐5p Inhibits the Proliferation and Migration of BC Cells

We analysed the expression levels of miR‐20a‐5p in BC cell lines (T24, J82, UMUC‐3 and SW780) and normal bladder epithelial cells (SV‐HUC‐1) using RT‐qPCR. The results showed that, compared to SV‐HUC‐1 cells, miR‐20a‐5p was significantly downregulated in all BC cell lines (T24, SW780, UMUC‐3 and J82) (Figure 6B). Subsequently, we overexpressed miR‐20a‐5p in the two BC cell lines (T24 and J82) with the lowest miR‐20a‐5p expression using miR‐20a‐5p mimics and confirmed the efficiency of overexpression by RT‐qPCR (Figure 6C). Compared to the negative control (NC) group, CCK‐8 assays demonstrated that miR‐20a‐5p overexpression significantly inhibited the viability of T24 and J82 cells (Figure 6D). To further investigate the effect of miR‐20a‐5p on cell migration, we performed a wound healing assay, which revealed that miR‐20a‐5p overexpression significantly suppressed the migratory capacity of T24 and J82 cells (Figure 6E). In addition, flow cytometry analysis showed that miR‐20a‐5p overexpression significantly reduced the proportion of J82 and T24 cells in the G2/M phase (Figure 6F), indicating that miR‐20a‐5p inhibits cell division and growth by interfering with cell cycle progression. These experimental findings collectively confirm the tumour‐suppressive role of miR‐20a‐5p in BC.

## 4 Discussion

BC, as a major public health concern, has a complex and varied pathogenesis that significantly contributes to its unsatisfactory treatment outcomes. While modern medicine has achieved certain advancements in BC treatment, including surgery, chemotherapy and immunotherapy, the high heterogeneity and recurrence of tumours continue to present serious challenges to patient prognosis [25]. Therefore, a deeper understanding of the molecular mechanisms of BC, particularly the identification of new biomarkers and therapeutic targets, is crucial. Studies have demonstrated that molecular biomarkers can be utilised not only for early tumour screening but are also closely linked to treatment decisions and patient prognosis [26]. Thus, the search for new biomarkers and targeted therapeutic strategies can help further improve clinical outcomes for BC patients.

This study systematically integrates bioinformatics analysis with experimental validation, revealing for the first time the critical role of miR‐20a‐5p in BC. The results show that miR‐20a‐5p is significantly downregulated in BC tissues, and survival analysis indicates a correlation between its low expression and poor patient prognosis. This finding not only highlights the value of miR‐20a‐5p as a potential biomarker but also provides clinicians with a new prognostic assessment tool. Considering the unique expression pattern of miR‐20a‐5p and its impact on patient survival and recurrence risk, future research should explore the potential of incorporating miR‐20a‐5p into clinical testing to facilitate personalised treatment. Additionally, we observed that miR‐20a‐5p expression levels are significantly associated with multiple clinicopathological parameters, such as T stage and tumour grade, suggesting that miR‐20a‐5p may play an important role in the early diagnosis and treatment of BC.

During tumorigenesis and progression, the expression of miRNAs is regulated by a complex network involving multiple signalling pathways and tumour subtype‐specific variations. miRNAs modulate the expression of their target mRNAs by binding to them, thereby influencing biological functions such as cellular processes, proliferation and migration [27, 28]. In this study, we identified 76 target genes regulated by miR‐20a‐5p in BC, including KPNA2, which has been reported to function as an oncogene in various cancers. KPNA2 (also known as Rch1 or hSRP1) plays a critical role in cell signal transduction and nucleocytoplasmic transport, and it has been shown to act as an oncogene involved in the occurrence and development of various cancers [29, 30]. However, its exact function remains incompletely understood. Previous studies have shown that KPNA2 enhances extracellular matrix (ECM) signalling by mediating the nuclear translocation of CREB3L1, thereby promoting the growth and metastasis of anaplastic thyroid carcinoma [31]. Furthermore, KPNA2 has been reported to facilitate hepatocellular carcinoma malignancy by mediating KDM4A‐SA1‐induced activation of the AKT pathway [32]. Additionally, KPNA2 promotes gallbladder cancer progression by regulating the nuclear localization of E2F1 and E2F7 [23]. Our previous study demonstrated that KPNA2 is upregulated in BC tissues and is associated with poor prognosis [33]. In this study, a dual‐luciferase reporter assay further confirmed that miR‐20a‐5p can directly regulate KPNA2 expression. This finding reinforces the role of KPNA2 as a potential therapeutic target and reveals the mechanism by which miR‐20a‐5p exerts its inhibitory effect in BC.

To comprehensively elucidate the role of miR‐20a‐5p in BC, we conducted a series of in vitro experiments. The results indicated that the expression of miR‐20a‐5p was significantly reduced in BC cell lines, and its overexpression effectively inhibited the proliferation and migration of BC cells, further underscoring its regulatory effect on KPNA2. Additionally, KEGG and GO enrichment analyses demonstrated that miR‐20a‐5p expression is closely related to cell cycle regulation, while GSEA analysis revealed a significant association with the autophagy process. These findings suggest that miR‐20a‐5p may suppress BC progression by blocking the cell cycle and inducing tumour cell autophagy, although the specific mechanisms require further experimental validation.

In summary, this study systematically investigated the role of miR‐20a‐5p in BC, revealing its significance as a potential biomarker and therapeutic target. Moreover, its low expression is clearly associated with poor prognosis in patients. By regulating KPNA2, miR‐20a‐5p not only affects cancer cell proliferation and migration but may also influence tumour progression through interactions with multiple signalling pathways. However, this study has certain limitations. The absence of clinical follow‐up data restricts the evaluation of miR‐20a‐5p's practical clinical application as a prognostic marker. Future research should consider conducting larger scale, multicentre clinical trials to more systematically assess the role and potential clinical value of miR‐20a‐5p in BC. Additionally, in‐depth in vivo and in vitro experiments will help clarify the specific mechanisms of miR‐20a‐5p and its functions in different BC subtypes.

Through these efforts, we aim to deepen the understanding of the molecular mechanisms underlying BC and provide an experimental foundation and theoretical support for potential targeted therapy strategies in the future. Although current research on miR‐20a‐5p is still in the exploratory stage, with the accumulation of relevant data, its role as a potential biomarker will become increasingly clear.

## Author Contributions

**Shuai Ye:** data curation (lead), methodology (lead), resources (lead), software (lead), writing – original draft (lead). **Cen Liufu:** formal analysis (lead), methodology (supporting), writing – original draft (equal). **Cong Yin:** investigation (lead), software (supporting), writing – review and editing (equal). **Tao Zhu:** formal analysis (supporting), supervision (supporting), validation (lead). **Jinqing He:** data curation (supporting), methodology (supporting). **Yuanyuan Tian:** formal analysis (supporting), validation (supporting). **Yan Wang:** funding acquisition (equal), supervision (lead), writing – review and editing (equal). **Bentao Shi:** funding acquisition (lead), supervision (equal), writing – review and editing (equal).

## Conflicts of Interest

The final version submitted has been reviewed and approved by all authors of this research. The authors confirm that there are no conflicts of interest.

## Data Availability

The datasets generated and analysed during the current study are available in the GEO (https://www.ncbi.nlm.nih.gov/geo/), TCGA (https://portal.gdc.cancer.gov). Further inquiries can be directed to the corresponding authors.
